# Supplementary figures and images for: Transcriptome analysis of Sonneratia caseolaris seedlings under chilling stress
Source: PeerJ. 2021 Jun 3;9:e11506. doi: 10.7717/peerj.11506 (PMC8180195; doi:10.7717/peerj.11506)

**A**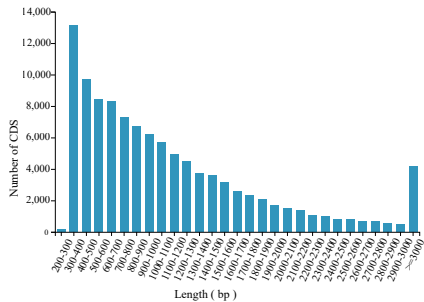**B**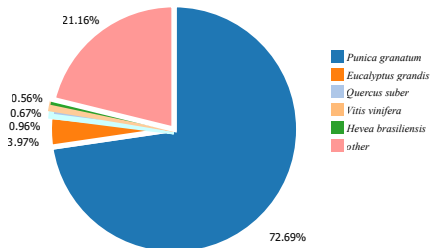**C**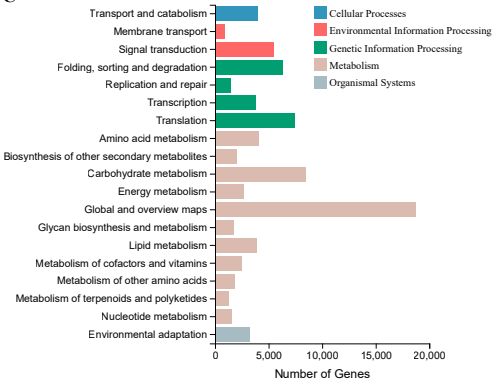**D**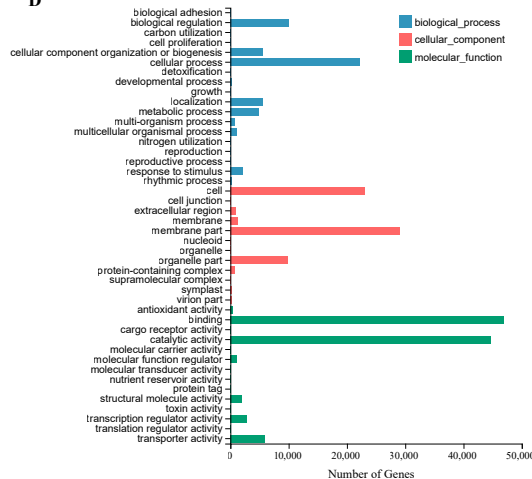

Supplement: Supplemental Information 1 — (A) Distribution of unigene lengths in Sonneratia caseolaris. (B) Main species distribution of S. caseolaris unigenes. (C) Pathway annotation of unigenes based on KEGG categorization (D) Functional annotation [file peerj-09-11506-s001.pdf]

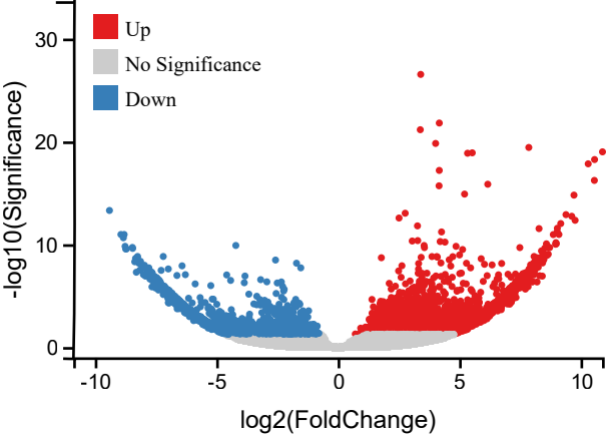

Supplement: Supplemental Information 2 [file peerj-09-11506-s002.pdf]
