# Supplementary material for: Transcriptome analysis of Sonneratia caseolaris seedlings under chilling stress
Source: PeerJ. 2021 Jun 3;9:e11506. doi: 10.7717/peerj.11506 (PMC8180195; doi:10.7717/peerj.11506)
Supplement: Supplemental Information 3 [file peerj-09-11506-s003.docx]

| Gene Name | Description | ID | Primers | |
| --- | --- | --- | --- | --- |
| *CAT* | Catalase | Unigene30041_All | | F:CCACAGAGTTCACAGCCTGA |
|  |  |  |  | R: AACCGTTCAACTCCACCTTG |
| *TRR* | Thioredoxin reductase | CL355.Contig5_All | | F: ATTGACTGGCGCAGAGAAGT |
|  |  |  |  | R: TTTCGTGTCGGAGAAGACCT |
| *AP2* | AP2-like factor | CL934.Contig12_All | | F: GGAAGAGGAAAAAGCCAAGC |
|  |  |  |  | R: GGAGTTCTCGTCTCCTGCAC |
| *WRKY22* | WRKY transcription factor 22 | CL2285.Contig4_All | | F: TGTCGACGATCACAGGAGAC |
|  |  |  |  | R: TATCGCCGGAACTCTTATCG |
| *SUSY* | Sucrose-phosphate synthase | CL2626.Contig4_All | | F: ATCACCGGATTCGATGAGAC |
|  |  |  |  | R: CGTTTTGCTTTCCTTTGAGC |
| *DOX* | Alpha-dioxygenase | CL4125.Contig8_All | | F: CTTCAGCTGTGATTGCCAAA |
|  |  |  |  | R: AGTCCCACCAATCCACTCAG |
| *ACAA1* | Acetyl-coa acyltransferase 1 | CL10456.Contig6_All | | F: CGGAGGTGGGGGATATAGTT |
|  |  |  |  | R: GCCAATCGCATCTACAGTCA |
| *SSY* | Sucrose synthase | CL14150.Contig25_All | | F: TCCTCATCGTGACTCGTCTG |
|  |  |  |  | R: GCAACATCCTCGGTGTACCT |
| *MYBP* | Transcription factor MYB, plant | CL1346.Contig5_All | | F: TAGGTCTCCGGATGAGATGG |
|  |  |  |  | R: ACCGTCCACCACCAGTAAAC |
| *ETR* | Ethylene receptor | CL2232.Contig4_All | | F: TGTGGCTGCTATCAGAATGC |
|  |  |  |  | R: CCTCAAGCTCTTCCCTTGTG |
| *ABF* | ABA responsive element binding factor | CL4581.Contig8_All | | F: AGCAACTCTGGCTTTTGCAT |
|  |  |  |  | R: TGCAGGTGATAACTGGCTTG |
| *PP2C* | Protein phosphatase 2C | CL9193.Contig2_All | | F: ACGGGATATGGAAGATGCTG |
|  |  |  |  | R: CGGTCCATCCTTGTGAAACT |
| *ACTIN* | ACTIN |  | | F: GAAAGAGGTCGTGGTGGAGA |
|  |  |  |  | R: AGGAAGCTAACCACGCAATG |
