# Supplementary material for: Transcriptome analysis of Sonneratia caseolaris seedlings under chilling stress
Source: PeerJ. 2021 Jun 3;9:e11506. doi: 10.7717/peerj.11506 (PMC8180195; doi:10.7717/peerj.11506)
Supplement: Supplemental Information 4 [file peerj-09-11506-s004.docx]

| Sample | Total Clean Bases(Gb) | Total Raw Reads (M) | Total Clean Reads (M) | Total Number | Mean Length | N50 | N90 | GC (%) |
| --- | --- | --- | --- | --- | --- | --- | --- | --- |
| CK_1 | 6.38 | 45.57 | 42.52 | 71,593 | 1,479 | 2,216 | 784 | 45.36 |
| CK_2 | 6.36 | 45.57 | 42.42 | 63,172 | 1,473 | 2,183 | 784 | 45.44 |
| CK_3 | 6.66 | 47.73 | 44.38 | 84,194 | 1,241 | 2,128 | 522 | 44.69 |
| CT_1 | 6.64 | 47.73 | 44.28 | 111,265 | 1,350 | 2,316 | 587 | 43.43 |
| CT_2 | 6.63 | 47.73 | 44.22 | 63,830 | 1,279 | 2,124 | 569 | 45.66 |
| CT_3 | 6.66 | 47.73 | 44.39 | 96,272 | 1,729 | 2,666 | 939 | 44.71 |
| All-Unigene | 39.33 | 280.46 | 262.21 | 168,473 | 1,762 | 2,849 | 937 | 43.94 |
