# Supplementary material for: Transcriptome analysis of Sonneratia caseolaris seedlings under chilling stress
Source: PeerJ. 2021 Jun 3;9:e11506. doi: 10.7717/peerj.11506 (PMC8180195; doi:10.7717/peerj.11506)
Supplement: Supplemental Information 5 [file peerj-09-11506-s005.docx]

| Database | Number of unigenes | Percentage |
| --- | --- | --- |
| NR | 116,619 | 69.22% |
| NT | 85,761 | 50.90% |
| Swissprot | 90,660 | 53.81% |
| KEGG | 94,737 | 56.23% |
| KOG | 100,382 | 59.58% |
| Pfam | 96,869 | 57.50% |
| GO | 92,876 | 55.13% |
| Intersection | 48,858 | 29.00% |
| Overall | 125,360 | 74.41% |
